# *Rhodococcus folensis* sp. nov., an orange-red-pigmented bacterium from mining soil

**DOI:** 10.1038/s41598-026-58503-0

**Published:** 2026-06-21

**Authors:** Aleyna Nalcaoglu Senocak, Omer Faruk Ozturk, Miray Kilic, Ersan Bektas, Ali Osman Belduz, Kadriye Inan Bektas

**Affiliations:** 1 Faculty of Science, Department of Molecular Biology and Genetics, Karadeniz Technical University, 61080 Trabzon, Türkiye; 2 Graduate School of Natural and Applied Science, Department of Molecular Biology and Genetics, Karadeniz Technical University, Trabzon, 61080 Turkey; 3 Espiye Vocational School, Giresun University, Giresun, Turkey; 4 Faculty of Science, Department of Biology, Karadeniz Technical University, 61080 Trabzon, Türkiye

**Keywords:** Polyphasic taxonomy, genome-based species delineation, *Rhodococcus*, carotenoid, antioxidant activity, HIV-RT inhibition, Biotechnology, Microbiology

## Abstract

Mining-impacted environments represent chemically complex ecosystems that may harbor metabolically versatile and pigment-producing microorganisms. During a survey of pigment-producing bacteria from abandoned mining soil in Trabzon, Türkiye, a red-pigmented strain, designated FMA22^T^, was isolated and characterized using a polyphasic taxonomic approach. 16 S rRNA gene sequence analysis placed the strain within the genus *Rhodococcus*, showing the highest similarity to *R. corynebacterioides* DSM 20,151^T^ (99.57%), *R. kroppenstedtii* DSM 44908ᵀ (99.06%) and *R. trifolii* T8^T^ (98.96%). The strain was Gram-stain-positive, aerobic and non-motile, and grew at 4–40 °C. Polar lipids included phosphatidylethanolamine, diphosphatidylglycerol, phosphatidylinositol, phosphatidylinositol mannoside, phosphatidylcholine, five unidentified glycolipids, four unidentified lipids, one unidentified phospholipid and one unidentified phosphoglycolipid; MK-8(H2) was the major respiratory quinone. Major fatty acids were C18:1 ω9c, summed feature 3 (C16:1 ω7c/C16:1 ω6c) and C16:0. ANI and dDDH values with the closest relatives were below 76.8% and 20.5%, respectively. The draft genome (4.23 Mb; 67.2 mol% G + C; 4,106 CDSs) harbors a terpene-associated carotenoid cluster containing crtB, crtI and crtY. The orange-red pigment (λmax = 475 nm) showed antioxidant activity (DPPH SC₅₀ = 5.38 mg mL⁻¹; FRAP = 4.34 µmol TE g⁻¹) and weak but measurable HIV-1 reverse transcriptase inhibition (IC₅₀ = 22 mg mL⁻¹). These data support the proposal of *Rhodococcus folensis* sp. nov., with FMA22ᵀ (= LMG 34144ᵀ = DSM 120048ᵀ) as the type strain.

**Supplementary Information:**

The online version contains supplementary material available at https://doi.org/10.1038/s41598-026-58503-0.

## Introduction

The genus *Rhodococcus*, belonging to the phylum *Actinomycetota*, was first proposed by Zopf in 1891, with *Rhodococcus rhodochrous* designated as the type species, and was later validated in IJSEM^1,2^. At the time of writing, this genus comprised 59 species with validly published names, as listed in the List of Prokaryotic Names with Standing in Nomenclature (LPSN, https://lpsn.dsmz.de, accessed on 17 February 2026)^3^. The genus *Rhodococcus* comprises aerobic, Gram-positive, non-sporulating bacteria that are widely distributed in terrestrial and contaminated environments. Members of this genus are well recognized for their remarkable metabolic versatility, including hydrocarbon degradation, xenobiotic transformation, and the production of structurally diverse secondary metabolites^4,5^. Genome-based analyses have further highlighted the extensive biosynthetic gene cluster diversity within *Rhodococcus*, supporting its relevance for environmental biotechnology and natural product discovery^6^. Actinobacterial genomes, including those of *Rhodococcus*, are increasingly recognized as compounds of potential biotechnological interest^7^.

*Rhodococcus* species have been isolated from diverse ecological niches, including hydrocarbon-contaminated soils, heavy metal-rich sediments, and industrially impacted sites^4,5^. Abandoned mining areas represent chemically complex ecosystems characterized by metal enrichment, oxidative stress conditions, nutrient limitation, and fluctuating physicochemical parameters. These selective pressures may favor microorganisms capable of producing protective metabolites, including antioxidant pigments, making mining-impacted habitats promising yet underexplored reservoirs of pigment-producing bacteria.

Carotenoids and other pigmented microbial metabolites contribute to cellular redox homeostasis by scavenging reactive oxygen species (ROS), thereby enhancing stress tolerance and membrane stability^8^. Beyond ecological adaptation, microbial carotenoids have attracted increasing attention for applications in food, nutraceutical, cosmetic, and biomedical fields due to their antioxidant and bioactive properties^9^. In parallel, there has been growing interest in natural compounds with antiviral potential, particularly those targeting essential viral enzymes involved in genome replication. Among these targets, reverse transcriptase represents a biologically meaningful enzyme system because its activity is directly associated with the conversion of viral RNA into DNA, a critical and early step in the replication cycle of retroviruses. Therefore, inhibition of reverse transcriptase provides a direct and measurable enzyme-level indication of potential interference with viral replication mechanisms.

Human immunodeficiency virus (HIV), the causative agent of acquired immunodeficiency syndrome (AIDS), remains a major global public health concern. HIV-1 Reverse Transcriptase (HIV-1 RT) is indispensable for viral genome replication and constitutes a primary target of antiretroviral therapy. Although synthetic reverse transcriptase inhibitors are widely used, the emergence of drug-resistant viral strains and therapy-associated adverse effects necessitate continued exploration of structurally diverse natural inhibitors^10^. For this reason, HIV-1 RT has frequently been used in natural product research as a defined enzymatic target for preliminary screening, particularly when the tested material consists of complex extracts or secondary metabolite mixtures whose individual active components have not yet been purified. This approach allows the biological relevance of crude extracts to be assessed at the molecular level before further purification and structural characterization.

Several bacterial pigments have been investigated for antiviral activities in different experimental systems^9,11,12^. Notably, violacein and deoxyviolacein derived from *Janthinobacterium* species have been evaluated for direct in vitro inhibition of recombinant HIV-1 RT^13^. Nevertheless, the interaction of pigments derived from *Rhodococcus* species with HIV-1 RT has not been comprehensively evaluated, and data in this specific context remain unavailable. Accordingly, the present study focused on HIV-1 RT inhibition not as a comprehensive antiviral evaluation but rather as a rational enzyme-based preliminary screening strategy to determine whether the carotenoid-type pigment extract produced by strain FMA22^T^ exhibits detectable interaction with a key viral replication enzyme. This focus was further justified by the crude nature of the pigment extract and by the absence of previous data on HIV-1 RT inhibition by *Rhodococcus*-derived pigments.

In the present study, an orange-red-pigmented bacterial strain was isolated from soil collected at an abandoned mine site in Vakfıkebir, Türkiye. A polyphasic taxonomic approach was subsequently applied to characterise strain FMA22^T^, and the results of the taxonomic analysis indicate that strain FMA22^T^ represents a novel species of the genus *Rhodococcus*, for which the name *Rhodococcus folensis* sp. nov. is proposed. Beyond its taxonomic novelty, the strain produces a distinctive orange-red pigment, which was extracted and spectroscopically characterized. The antioxidant capacity of the pigment was evaluated using the DPPH radical scavenging assay, and its inhibitory activity against HIV-1 reverse transcriptase was assessed. Collectively, these findings expand the taxonomic diversity of the genus *Rhodococcus* and highlight mining environments as sources of pigment-producing bacteria with antioxidant properties and detectable enzyme-level interaction with HIV-1 RT.

## Materials and methods

### Strain isolation, ecological background and culture conditions

Strain FMA22ᵀ was isolated from a soil sample collected at an abandoned mining site in Kalınçam village, Vakfıkebir district, Trabzon, Türkiye, a locality historically referred to as “Fol” (40°53’12.0” N, 39°17’26.6” E). The site corresponds to a former mining exploration area that is currently abandoned and not part of any protected zone, private property or an active mining operation; therefore, no specific permit was required for soil sampling. The collected soil sample was air-dried in a sterile petri dish at room temperature for 14 days. After the drying period, the soil sample was suspended in Ringer’s solution, shaken for 30 min at 28 °C, and subsequently heat-treated at 60 °C for 20 min. Serial dilutions were prepared, and 200 µl aliquots from the 10⁻⁴ dilution were spread onto R2A agar supplemented with antibiotics (cycloheximide, 50 µg/ml; nalidixic acid, 10 µg/ml; and nystatin, 50 µg/ml). This treatment was applied to reduce the abundance of fast-growing or non-target microorganisms and to facilitate the selective isolation of actinobacteria, rather than specifically targeting pigment-producing bacteria^14^. The R2A medium contained (per litre): yeast extract, 0.5 g; peptone, 0.5 g; casamino acids, 0.5 g; glucose, 0.5 g; starch, 0.5 g; sodium pyruvate, 0.3 g; K₂HPO₄, 0.3 g; MgSO₄·7 H₂O, 0.5 g; agar (Difco), 18 g; distilled water, 1000 ml (pH 7.2). Plates were incubated at 28 °C for 10 days. An orange-red colony was picked and inoculated into Nutrient Broth to obtain a pure culture. Colony morphology and microscopy were used to assess the purity of the isolate. Strain FMA22ᵀ was routinely cultivated on Nutrient Agar and preserved at -80 °C in Nutrient Broth supplemented with glycerol (15%, v/v).

### 16 S rRNA gene sequencing and phylogenetic analyses

Genomic DNA of strain FMA22ᵀ was obtained from cells grown aerobically in nutrient broth at 25 °C for 18–24 h. DNA extraction was carried out using the Wizard^®^ Genomic DNA Purification Kit (Promega, Madison, WI, USA) according to the manufacturer’s instructions. The nearly complete 16 S rRNA gene was amplified by PCR using the universal bacterial primer pair UNI16S-L and UNI16S-R^15^. Amplified products were purified and cloned into the pGEM^®^-T Easy vector system, followed by transformation into Escherichia coli JM101 competent cells. Recombinant plasmids were recovered from selected transformants and submitted for Sanger sequencing (Macrogen Europe, Amsterdam, The Netherlands). Bidirectional sequencing reads were assembled to obtain a consensus 16 S rRNA gene sequence after quality trimming and removal of primer regions. The resulting sequence was compared with reference sequences of validly published type strains using the EzBioCloud server (www.ezbiocloud.net) to identify phylogenetically related taxa^16^. Multiple sequence alignment was performed using BioEdit, considering only homologous and overlapping regions shared among the sequences. Evolutionary distances were calculated based on the Kimura two-parameter substitution model^17^. Phylogenetic reconstructions were conducted using MEGA X software employing the neighbor-joining^18^, maximum-likelihood^19^, and maximum-parsimony^20^ algorithms. The robustness of tree topologies was evaluated by bootstrap analysis with 1000 replicates.

### Whole-genome sequencing and genomic analyses

Whole-genome sequencing of strain FMA22ᵀ was performed by RefGen (Türkiye) using the Illumina HiSeq 2500 platform, generating 2 × 250 bp paired-end reads. Raw sequencing data were quality-checked and assembled de novo using the SPAdes algorithm implemented through the PATRIC web-based server (https://patricbrc.org/)^21^. The resulting draft genome assembly was assessed for quality metrics, including completeness and contamination, using the CheckM2 pipeline^22^. Automated functional annotation was carried out using the RAST server and SEED Viewer (version 2.0), which assigns predicted coding sequences to subsystem-based functional categories^23^. To clarify the phylogenetic placement of strain FMA22ᵀ within the genus *Rhodococcus*, genome-based phylogenomic analyses were conducted. Closely related taxa were selected based on 16 S rRNA gene sequence similarity results obtained from the EzBioCloud database, and all type strains with publicly available genome assemblies in the NCBI GenBank database were included in downstream analyses. A high-resolution phylogenomic tree was reconstructed using the UBCG2 (up-to-date bacterial core gene) pipeline^24^, which infers evolutionary relationships based on a concatenated alignment of 81 conserved bacterial core genes. In parallel, whole-genome phylogenetic inference and intergenomic distance calculations were performed using the type (Strain) genome server (TYGS) (https://tygs.dsmz.de/)^25^, which applies the genome BLAST distance phylogeny (GBDP) method.

Genome-scale relatedness between strain FMA22ᵀ and phylogenetically related type strains was further evaluated using multiple indices. Average nucleotide identity (ANI) values were calculated through the EzBioCloud ANI calculator (https://www.ezbiocloud.net/tools/ani)^26^, while average amino acid identity (AAI) was determined using the Enveomics platform (https://enve-omics.gatech.edu/)^27^. Digital DNA–DNA hybridization (dDDH) estimates were retrieved from the TYGS output, which implements the genome-to-genome distance calculator (GGDC) algorithm for in silico DNA relatedness assessment^28^.

Metabolic pathway reconstruction and functional categorization of genes were performed using the KEGG database (kyoto encyclopedia of genes and genomes) (https://www.genome.jp/kegg/)^29^. Secondary metabolite biosynthetic gene clusters were predicted using antiSMASH version 6.0 (https://antismash.secondarymetabolites.org/)^30^. Carbohydrate-active enzymes (CAZymes) were identified via the dbCAN3 meta server (http://bcb.unl.edu/dbCAN3)^31^, which integrates HMMER, DIAMOND, and Hotpep algorithms to assign genes to CAZyme families, including glycoside hydrolases (GHs), glycosyltransferases (GTs), carbohydrate-binding modules (CBMs), polysaccharide lyases (PLs), auxiliary activities (AAs), and carbohydrate esterases (CEs).

### Morphological, physiological and biochemical characterization

For comparative phenotypic analyses, the type strains *R. corynebacterioides* DSM 20,151^T^, *R. kroppenstedtii* DSM 44,908^T^, and *R. trifolii* T8^T^ were obtained from the Deutsche Sammlung von Mikroorganismen und Zellkulturen (DSMZ, Germany). The reference strains were cultivated under identical laboratory conditions to ensure consistency in all comparative experiments.

Cell morphology and staining properties were evaluated using conventional Gram staining procedures and verified by the KOH string test^32,33^. Motility was examined using semisolid agar supplemented with triphenyltetrazolium chloride (TTC), where diffusion from the inoculation point was interpreted as positive motility following incubation^34^. Anaerobic growth capability was assessed by incubating inoculated nutrient agar plates in anaerobic jars at 25 °C for 72 h. The growth characteristics of strain FMA22^T^ were investigated on nutrient agar (NA), Luria-Bertani (LB) agar, and tryptic soy agar (TSA). Temperature tolerance was determined by incubating cultures in nutrient broth at temperatures ranging from 4 °C to 50 °C at 5 °C increments. The pH growth range was evaluated in nutrient broth adjusted between pH 4.0 and 10.5 (intervals of 0.5 units), while NaCl tolerance was tested in nutrient broth containing 0–5% (w/v) NaCl. All tolerance experiments were performed at 25 °C and monitored over a period of up to seven days.

Oxidase activity was determined using tetramethyl-p-phenylenediamine as the electron donor substrate, whereas catalase production was assessed by monitoring bubble formation following exposure to 3% hydrogen peroxide^35^. Additional biochemical and metabolic traits of strain FMA22^T^ and the reference strains were characterized using the API 20E, API 50CH, and VITEK 2 GP identification systems (bioMérieux), in accordance with the manufacturer’s protocols.

### Chemotaxonomic analyses

Chemotaxonomic features of strain FMA22^T^ were determined by examining cellular fatty acid composition, polar lipid profile, and respiratory quinone content. For comparative purposes, the reference strains *R. corynebacterioides* DSM 20,151^T^, *R. kroppenstedtii* DSM 44,908^T^, and *R. trifolii* T8^T^ were cultivated under the same experimental conditions. For fatty acid analysis, biomass was obtained from cultures grown on nutrient agar plates at 25 °C for 48 h under identical and standardized cultivation conditions to ensure comparability of fatty acid profiles. Approximately 40 mg of cells were processed to obtain fatty acid methyl esters (FAMEs) using the standard protocol of the Sherlock Microbial Identification System (MIDI; Microbial ID Inc., Newark, DE, USA), which includes saponification, methylation, and extraction steps, followed by gas chromatographic analysis^36^.

Polar lipids were extracted from freeze-dried cell material (approximately 100 mg) using organic solvents and separated by two-dimensional thin-layer chromatography (TLC). In the first dimension, a chloroform-methanol-water (65:25:4, v/v) solvent system was employed, followed by separation in a second dimension using chloroform-methanol-acetic acid-water (80:12:15:4, v/v). Lipid classes were detected using specific staining reagents: molybdophosphoric acid for total lipids, molybdenum blue for phospholipids, ninhydrin for aminolipids, and α-naphthol for glycolipids^37^. Respiratory quinones were extracted from freeze-dried cells and analysed by high-performance liquid chromatography (HPLC) following established procedures for bacterial quinone profiling^38^. The isomeric form of diaminopimelic acid was determined according to the method described by Becker et al. (1964)^39^, and whole-cell sugar composition was analyzed following the protocol of Lechevalier (1968)^40^.

### Pigment production and extraction

Strain FMA22^T^ was cultured in nutrient broth at 20 °C under continuous shaking (150 rpm) for 72 h to enhance pigment synthesis. Following incubation, cells were harvested by centrifugation and the intensely pigmented biomass was collected. Pigment extraction was performed using methanol as the extraction solvent^41^. The harvested cell pellet was resuspended in methanol at a solvent-to-biomass ratio of 2:1 (v/w) and thoroughly mixed by vortexing to achieve complete homogenization. The suspension was subsequently incubated at 60 °C for 15 min to promote efficient release of intracellular pigments. After centrifugation, the colored supernatant was carefully recovered and passed through a 0.22 μm membrane filter to remove residual cell debris.

The absorbance spectrum of the methanolic extract was recorded over the full UV–visible range using a Cary 100 Bio UV–Vis spectrophotometer (Varian) to determine characteristic absorption maxima. In parallel, genes potentially involved in carotenoid biosynthesis were identified through whole-genome annotation analyses.

### Evaluation of antioxidant activity

The antioxidant capacity of the pigment extract obtained from strain FMA22ᵀ was investigated using two independent spectrophotometric assays: DPPH radical scavenging activity and ferric reducing antioxidant power (FRAP). For the DPPH assay, methanolic solutions of the pigment extract were prepared at different concentrations^42^. Each solution was mixed with freshly prepared DPPH solution (0.004%, w/v, in methanol) and incubated at room temperature in the absence of light for 30 min. The decrease in absorbance was monitored at 517 nm using a UV-Vis spectrophotometer. Radical scavenging activity was expressed as percentage inhibition relative to the control. The half-maximal scavenging concentration (SC₅₀) was calculated from dose–response curves obtained by plotting inhibition percentages against extract concentrations. Trolox^®^ was employed as a reference antioxidant. All experiments were conducted in triplicate.

The ferric reducing antioxidant power of the extract was determined using the FRAP method^43^. The FRAP working solution was freshly prepared by mixing acetate buffer (0.3 M, pH 3.6), TPTZ solution (0.01 M in 0.04 M HCl), and FeCl₃·6 H₂O (0.02 M). An aliquot of the extract (100 µL) was added to 3 mL of FRAP reagent, and the reaction mixture was incubated at 25 °C for 4 min. The increase in absorbance was measured at 593 nm. Antioxidant capacity was quantified based on a Trolox calibration curve and expressed as µmol Trolox equivalents (TE) per gram of extract. All measurements were performed in triplicate.

### HIV-1 reverse transcriptase inhibition activity

The inhibitory effect of the pigment extract on HIV-1 reverse transcriptase (HIV-1 RT) activity was evaluated using a non-radioactive colorimetric ELISA-based assay kit (Roche Diagnostics, Germany). All experimental procedures were conducted in accordance with the manufacturer’s guidelines. The dried pigment extract was dissolved in 10% (v/v) DMSO to obtain a stock solution at a concentration of 150 mg/mL. The pigment extract was prepared at serial dilutions from the stock solution to obtain a concentration range suitable for inhibition analysis. Recombinant HIV-1 RT enzyme was diluted in lysis buffer and added to each reaction well at the recommended working concentration. The reaction mixture consisted of enzyme solution, reaction buffer, and diluted extract, and plates were incubated at 37 °C for 1 h to allow enzyme-inhibitor interaction. Following incubation, wells were washed to remove unbound components, and anti-digoxigenin-peroxidase conjugate was added according to the kit protocol. After a second incubation step and subsequent washing, ABTS substrate solution was applied to initiate color development. The reaction was allowed to proceed at room temperature, and absorbance was measured at 405 nm using a microplate reader, with a reference wavelength of 490 nm. Nevirapine was included as a positive control inhibitor, while wells lacking enzyme served as negative controls^43^. The percentage inhibition of HIV-1 RT activity was calculated relative to control reactions. The half-maximal inhibitory concentration (IC₅₀) was determined from dose-response curves generated by plotting inhibition percentages against extract concentrations. All experiments were performed in triplicate.

## Results and discussion

### Microbial Isolation

Cultivation of soil samples obtained from the abandoned mining site yielded a diverse array of microbial colonies exhibiting distinct morphological characteristics. Among the recovered bacterial isolates, a broad pigmentation spectrum was observed, ranging from yellow and its various shades to orange and orange-red coloration. A total of eight morphologically distinct pigmented bacterial isolates representing this color diversity were selected and purified for further investigation. This chromatic diversity suggests that the mining soil harbors a pigment-rich microbial community. Among the pigmented isolates, strain FMA22ᵀ exhibited a distinct orange-red pigmentation and was the only isolate displaying this coloration. Colonies of strain FMA22ᵀ were circular, convex, and smooth-edged, and consistently maintained their pigmentation during repeated subcultivation. After 48 h incubation at 25 °C on nutrient agar, colonies reached approximately 0.5 mm in diameter. Cells were Gram-stain-positive, rod-shaped, and approximately 0.9–1.0 μm wide and 1.0–1.1 μm long. Representative colony morphology images of strain FMA22ᵀ and the compared reference strains are provided in Fig. S1.

The distinctive orange-red pigmentation of strain FMA22ᵀ, which remained stable during repeated subcultivation, rendered it phenotypically distinguishable and provided the initial basis for its detailed characterization. The occurrence of a pigmentation gradient from yellow to orange-red within the same habitat may reflect adaptive strategies associated with environmental stressors characteristic of mining sites, such as oxidative stress, heavy metal exposure, and fluctuating physicochemical conditions^44–46^. This ecological context provided additional rationale for the detailed taxonomic investigation of strain FMA22ᵀ.

### 16 S rRNA gene phylogenetic analysis

**Fig. 1:**
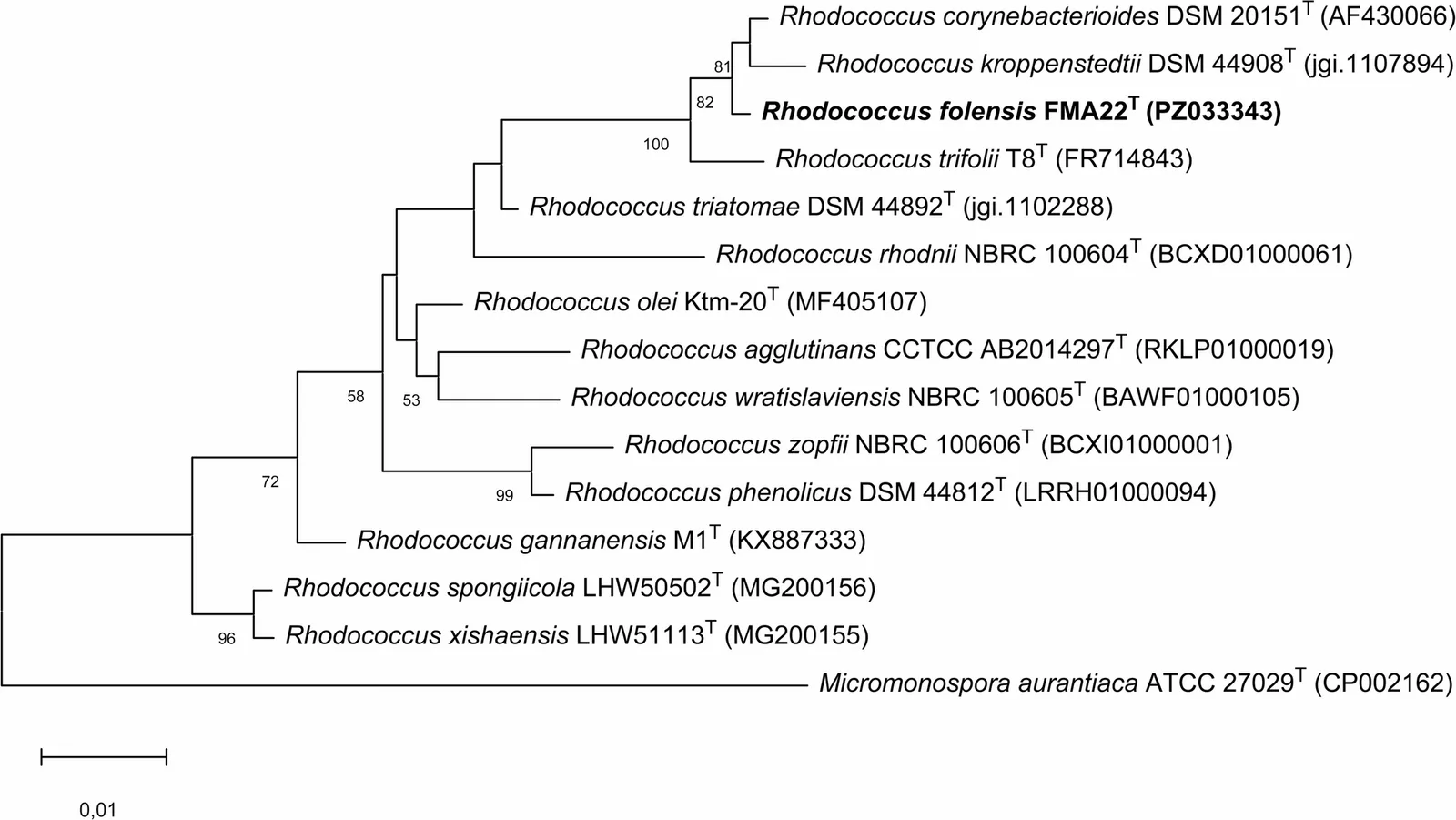
The relationship between strain FMA22^T^ and other related type strains are depicted in this maximum-likelihood phylogenetic tree, which is based on 16 S rRNA gene sequences. Bootstrap values exceeding 50% are indicated at the branch points. *Micromonospora aurantiaca* ATCC 27,029^T^ was used as an outgroup. Bar, 0.01 substitutions per nucleotide position.

The almost-complete 16 S rRNA gene sequences of the selected pigmented isolates were obtained and subjected to comparative sequence and phylogenetic analyses. Based on 16 S rRNA gene sequence comparisons, the eight selected isolates were affiliated with different bacterial genera, including *Rhodococcus* (FMA22ᵀ), *Micrococcus* (FMAX), *Pseudonocardia* (FMA9), *Plantibacter* (UGA11), *Williamsia* (FMA23), *Exiguobacterium* (UGA12), *Staphylococcus* (FMA1), and *Pseudomonas* (UGA5) (Table S1; Fig. 1; Figs. S2-S8). These results confirmed that the pigmented colonies recovered from the mining soil represented a taxonomically diverse bacterial assemblage rather than multiple isolates belonging to a single genus.

Among these isolates, FMA22ᵀ was notable because it was the only isolate exhibiting a distinct orange-red colony pigmentation. In addition, although its 16 S rRNA gene sequence showed high similarity to its closest type strain, the 16 S rRNA gene-based phylogenetic tree suggested a relatively distinct placement within the closely related *Rhodococcus* lineage. Therefore, FMA22ᵀ was prioritized for detailed taxonomic investigation, with genome-based analyses subsequently used to clarify its precise species-level status.

The almost-complete 16 S rRNA gene sequence of strain FMA22^T^ (1441 bp) was obtained and deposited in GenBank (accession number: PZ033343). Comparative analysis using the EzBioCloud database confirmed its affiliation with the genus *Rhodococcus*. Pairwise sequence similarity analysis revealed that strain FMA22^T^ shared the highest similarity with *R. corynebacterioides* DSM 20,151^T^ (99.57%), followed by *R. kroppenstedtii* DSM 44,908^T^ (99.06%) and *R. trifolii* T8^T^ (98.96%). Sequence similarities to other members of the genus were comparatively lower.

Phylogenetic reconstruction based on the maximum-likelihood (ML) algorithm showed that strain FMA22^T^ clustered within a strongly supported clade together with *R. corynebacterioides* DSM 20,151^T^, *R. kroppenstedtii* DSM 44,908^T^, and *R. trifolii* T8^T^ (bootstrap value: 100%) (Fig. 1). The same clustering topology was consistently recovered using neighbor-joining (NJ) and maximum-parsimony (MP) methods, both supported by 100% bootstrap values, indicating a stable phylogenetic placement of the isolate (Figs S9 and S10).

Although the 16 S rRNA gene sequence similarity between strain FMA22^T^ and *R. corynebacterioides* DSM 20,151^T^ exceeded the commonly used threshold for further species-level investigation, 16 S rRNA gene similarity alone is not sufficient for definitive species delineation, particularly among closely related bacterial taxa. Previous studies have emphasized that 16 S rRNA gene sequence analysis may have limited resolution at the species level and should be complemented by genome-based metrics such as ANI and dDDH for robust taxonomic conclusions^47,48^. Accordingly, higher-resolution genome-based analyses were performed to clarify the precise taxonomic position of strain FMA22^T^.

### Genome features and phylogenomic analysis

The draft genome sequence of strain FMA22^T^ comprised 54 contigs with a total length of 4,230,334 bp. The assembly showed high continuity, with an N50 value of 956,336 bp and an L50 of 2. Genome quality assessment using the CheckM2 pipeline indicated 100% completeness, together with high coarse (98.7%) and fine (97.6%) consistency scores, confirming the high quality of the assembly. The genomic DNA G + C content of strain FMA22ᵀ was 67.24%. Members of the genus *Rhodococcus* are known to possess relatively large, high-G + C genomes, typically ranging from approximately 3.9 to 10 Mb and 62–70% G + C content^49,50^. The genome size and G + C content of strain FMA22ᵀ fall within the reported genomic range of the genus. Genome annotation predicted 4,106 protein-coding sequences (CDSs), along with 45 tRNA genes and 6 rRNA genes. Functional annotation assigned a substantial proportion of CDSs to conserved protein families, whereas a subset was classified as hypothetical proteins. The nearly complete 16 S rRNA gene sequence obtained by Sanger sequencing (GenBank/EMBL/DDBJ accession number: PZ033343; 1441 bp) was compared with the corresponding full-length sequence extracted from the genome assembly (GenBank/EMBL/DDBJ accession number PZ092762; 1525 bp). Alignment of the overlapping regions revealed only a single nucleotide difference between the two sequences, confirming the consistency and reliability of the independently generated sequence data.

**Fig. 2:**
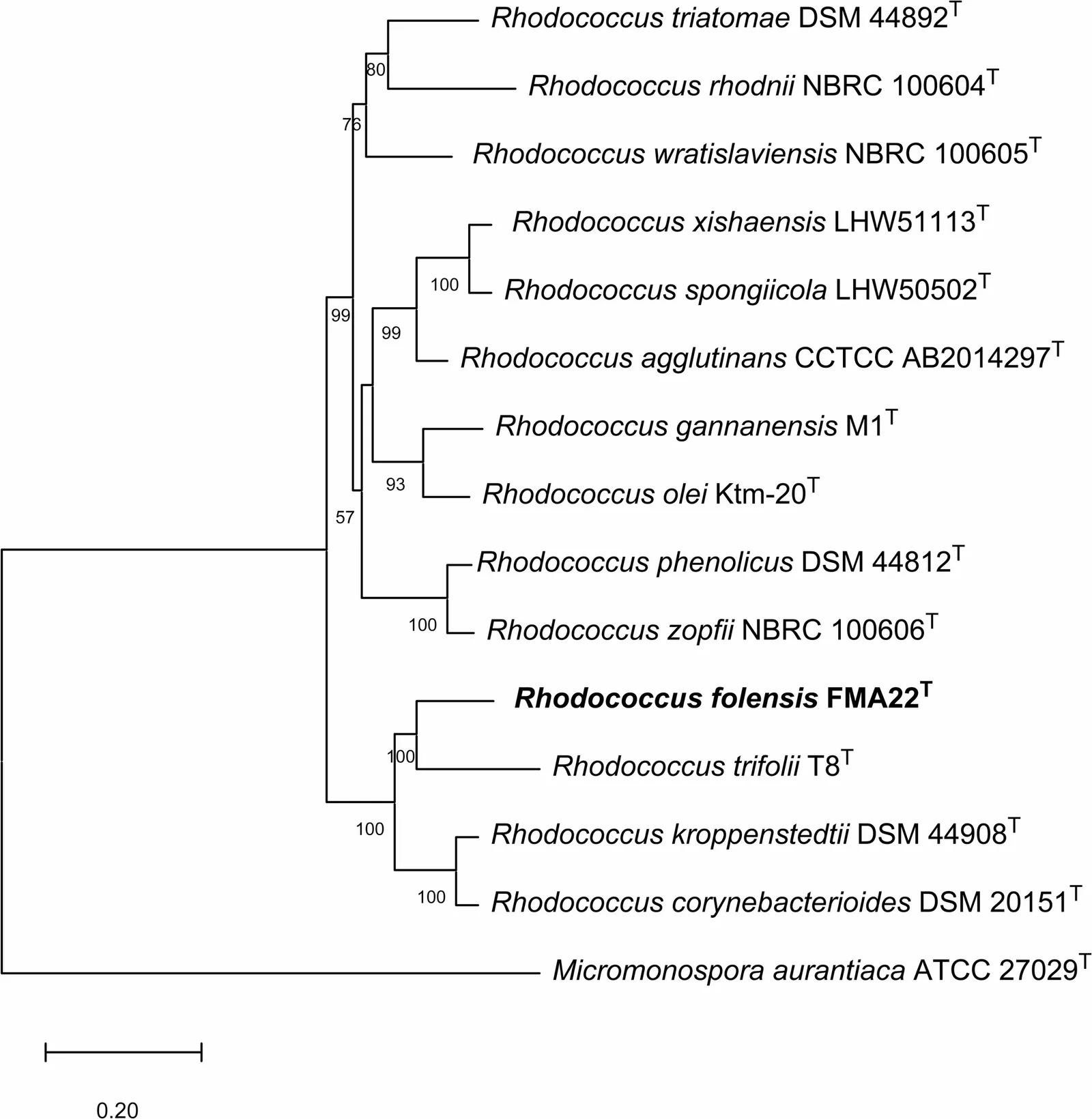
Phylogenomic tree constructed using UBCG2 based on publicly available whole-genome sequences of strain FMA22^T^ and related type strains.

Phylogenomic reconstruction based on the UBCG2 pipeline placed strain FMA22^T^ within the genus *Rhodococcus* (Fig. 2). In the UBCG2 tree, FMA22^T^ clustered in a strongly supported clade comprising *R. trifolii* T8ᵀ, *R. kroppenstedtii* DSM 44908ᵀ, and *R. corynebacterioides* DSM 20151ᵀ. This grouping was supported by high bootstrap values (100%), indicating robust phylogenomic coherence among these taxa. Within this clade, strain FMA22^T^ formed an independent branch, clearly separated from the neighboring type strains by distinct branch lengths. The overall topology demonstrated that this cluster is well differentiated from other *Rhodococcus* species included in the analysis. The genome-based phylogeny inferred using the TYGS platform recovered a congruent topology, placing FMA22^T^ within the same clade while maintaining its separate phylogenetic position (Fig. S11). The concordance between UBCG2 and TYGS analyses confirms the consistent phylogenomic placement of strain FMA22ᵀ within the genus *Rhodococcus*.

Genome relatedness between strain FMA22^T^ and its three closest phylogenetic relatives (*R. corynebacterioides* DSM 20151ᵀ, *R. kroppenstedtii* DSM 44908ᵀ, and *R. trifolii* T8ᵀ) was evaluated using average nucleotide identity (ANI), average amino acid identity (AAI), and digital DNA–DNA hybridization (dDDH) analyses (Table 1). The ANI value between strain FMA22^T^ and *R. corynebacterioides* DSM 20151ᵀ was 76.85%. Comparisons with R. *kroppenstedtii* DSM 44908ᵀ and R. *trifolii* T8ᵀ yielded ANI values of 76.42% and 75.86%, respectively. All values were substantially below the widely accepted 95–96% ANI threshold for species delineation, indicating clear genomic separation at the nucleotide level^51^. AAI values between strain FMA22^T^ and the same reference strains were 78.71% (*R. corynebacterioides* DSM 20151ᵀ), 78.64% (*R. kroppenstedtii* DSM 44908ᵀ), and 74.01% (*R. trifolii* T8ᵀ). These protein-level similarity values are also well below the 95% cutoff commonly used to define species boundaries, further supporting its distinct taxonomic status^52^. Consistent with ANI and AAI results, dDDH values obtained via the TYGS platform were markedly below the 70% species threshold^53^. The highest dDDH value was observed with *R. corynebacterioides* DSM 20151ᵀ (20.5%), followed by *R. kroppenstedtii* DSM 44908ᵀ (20.4%) and *R. trifolii* T8ᵀ (19.8%), confirming the genomic distinctiveness of strain FMA22^T^. Collectively, the nucleotide-, protein-, and genome distance–based metrics conclusively demonstrate that strain FMA22ᵀ represents a novel species within the genus *Rhodococcus*.

**Table 1:** ANIb, AAI and dDDH values between strain FMA22^T^ and related species of *Rhodococcus*.

	Genome Assembly	Strain FMA22^T^		
	Accession	ANIb	AAI	dDDH
*R. corynebacterioides* DSM 20151ᵀ	GCA_001646675.1	76.8	78.7	20.5
*R. kroppenstedtii* DSM 44908ᵀ	GCA_900111805.1	76.4	78.6	20.4
*R. trifolii* T8ᵀ	GCA_014635345.1	75.9	74.1	19.8

Comparative analysis of carbohydrate-active enzymes (CAZymes) revealed both conserved and species-level quantitative differences among strain FMA22^T^ and its closest phylogenetic relatives (*R. corynebacterioides* DSM 20151ᵀ, *R. trifolii* T8ᵀ, and *R. kroppenstedtii* DSM 44908ᵀ) (Table S2). Strain FMA22^T^ harbors 60 glycosyltransferases (GTs), 37 glycoside hydrolases (GHs), 5 carbohydrate-binding modules (CBMs), 12 carbohydrate esterases (CEs), and 7 auxiliary activity (AA) enzymes. The GT repertoire of FMA22ᵀ (60 genes) is comparable to that of *R. kroppenstedtii* DSM 44908ᵀ (57 genes) and *R. corynebacterioides* DSM 20151ᵀ (56 genes), but lower than that of *R. trifolii* T8ᵀ (69 genes). A similar trend was observed for GH enzymes: FMA22ᵀ encodes 37 GH genes, exceeding the count in *R. corynebacterioides* DSM 20151ᵀ (31 genes) and *R. kroppenstedtii* DSM 44908ᵀ (35 genes), yet remaining below that of *R. trifolii* T8ᵀ (42 genes). The number of CBM genes was largely conserved among the compared taxa, with FMA22^T^, *R. trifolii* T8ᵀ, and *R. corynebacterioides* DSM 20151ᵀ each harboring five CBM genes, while *R. kroppenstedtii* DSM 44908ᵀ contained six. This near-uniform distribution suggests a conserved capacity for carbohydrate-binding interactions within this phylogenetic cluster. Notable variation was observed in carbohydrate esterases (CEs) and auxiliary activity (AA) enzymes. Strain FMA22^T^ encodes 12 CE genes, a value higher than that of *R. corynebacterioides* DSM 20151ᵀ (10 genes) but markedly lower than those of *R. kroppenstedtii* DSM 44908ᵀ and *R. trifolii* T8ᵀ (21 genes each). Similarly, FMA22^T^ contains 7 AA genes, exceeding the count in *R. kroppenstedtii* DSM 44908ᵀ (5 genes) and *R. corynebacterioides* DSM 20151ᵀ (6 genes), yet remaining below that of *R. trifolii* T8ᵀ (11 genes). Overall, the CAZyme profile of strain FMA22^T^ shows a broadly similar distribution of major CAZyme classes to those of its closest phylogenetic relatives, while exhibiting moderate quantitative differences in specific enzyme families. These differences may contribute to variation in substrate utilization strategies and ecological specialization within this lineage.

Secondary metabolite biosynthetic gene clusters (BGCs) of strain FMA22^T^ were analyzed using antiSMASH 6.0 and compared with those of its closest phylogenetic relatives (*R. corynebacterioides* DSM 20151ᵀ, *R. trifolii* T8ᵀ and *R. kroppenstedtii* DSM 44908ᵀ) (Fig. S12, Table 2). The genome of strain FMA22ᵀ harbors multiple biosynthetic gene clusters, including NAPAA, NRPS, T1PKS, T3PKS, NRP-metallophore (erythrochelin), terpene, NRPS-like, ectoine, betalactone, and PKS-like clusters. Notably, a terpene biosynthetic cluster annotated as carotenoid was identified in strain FMA22^T^. This finding is consistent with the observed red pigmentation phenotype and supports the genomic basis of pigment production in this strain. In contrast, the terpene clusters detected in the related species showed similarity predominantly to isorenieratene-associated biosynthetic clusters in the MIBiG database. Comparative analysis further revealed differences in both gene organization and sequence similarity among the closely related species compared. The orange–red-pigmented strains FMA22ᵀ, *R. corynebacterioides* DSM 20151ᵀ, and *R. kroppenstedtii* DSM 44908ᵀ shared a similar carotenoid-related gene organization pattern consisting of polyprenyl synthetase (putative crtE)-crtI-α-(1→6)-mannopyranosyltransferase A-crtB, whereas the yellow-pigmented *R. trifolii* T8ᵀ displayed a different arrangement (crtB-α-(1→6)-mannopyranosyltransferase A-crtI-polyprenyl synthetase). Sequence-level comparisons of carotenoid-associated proteins also supported this divergence (Fig. S13). FMA22ᵀ exhibited moderate sequence similarity with related species for CrtI (72.6–77.0%), CrtB (68.9–74.7%), putative CrtE-related polyprenyl synthetase (69.7–71.2%), and α-(1→6)-mannopyranosyltransferase A (79.9–81.4%). In contrast, *R. corynebacterioides* DSM 20151ᵀ and *R. kroppenstedtii* DSM 44908ᵀ shared markedly higher similarities with each other, reaching 98.7%, 97.2%, 94.6%, and 100%, respectively. These findings indicate that carotenoid-related biosynthetic pathways are partially conserved among the compared closely related species, while strain FMA22ᵀ possesses distinct genomic characteristics compared with its nearest relatives, potentially contributing to its unique pigmentation phenotype. The presence of ectoine and erythrochelin clusters in strain FMA22^T^ further suggests adaptation to environmental stress conditions, as ectoine is commonly associated with osmotic stress tolerance, while siderophore-related metabolites such as erythrochelin are involved in iron acquisition under nutrient-limited conditions^54,55^. Additionally, the detection of mycosporine-related NRPS-like clusters (e.g., shinorine/4-deoxygadusol/mycosporine glycine) suggests a potential role in protection against oxidative or radiation-related stress, as mycosporine-like compounds are known for their photoprotective and antioxidant properties^56,57^. Comparative analysis revealed both conserved and strain-specific biosynthetic features. Core clusters such as NRPS, T3PKS, and ectoine were broadly distributed among the analyzed *Rhodococcus* species, whereas certain clusters, including RRE-containing, lanthipeptide-class-II/V, redox-cofactor, RiPP-like, and arylpolyene, were absent in strain FMA22^T^ but present in one or more related taxa. This differential distribution highlights genomic diversification among the compared closely related species and suggests ecological specialization among closely related species. Overall, the biosynthetic gene cluster profile of strain FMA22^T^ reflects metabolic features comparable to those observed in its closest phylogenetic relatives, while also displaying distinct elements that may contribute to its adaptation to the physicochemically challenging conditions of the abandoned mine environment.

**Table 2:** Secondary metabolites analysis of strain FMA22^T^ and its closest phylogenetic relatives. The biosynthetic gene clusters for secondary metabolites were analyzed using antiSMASH 6.0. (1) FMA22^T^; (2) *R. corynebacterioides* DSM 20151ᵀ; (3) *R. kroppenstedtii* DSM 44908ᵀ; (4) *R. trifolii* T8ᵀ.

NAPAA	1	2	3	4
	+	+	+	+
NRPS	+	+	+	+
T3PKS	+	+	+	+
NRP-metallophore	+	+	+	+
Terpene	+	+	+	+
T1PKS	+	-	+	+
NRPS-like	+	+	+	+
Ectoine	+	+	+	+
RRE-containing	-	+	-	-
lanthipeptide	-	+	-	+
Redox-cofactor	-	-	+	+
RİPP-like	-	-	-	+
Arylpolyene	-	-	-	+

Functional annotation of the FMA22ᵀ genome using the KEGG database revealed a broad and diverse metabolic repertoire (Table S3). A total of 1,539 genes were assigned to “global and overview maps,” reflecting a substantial metabolic capacity. Within primary metabolic categories, 245 genes were associated with carbohydrate metabolism, 240 with amino acid metabolism, and 166 with the metabolism of cofactors and vitamins. Additional functional assignments included 131 genes related to energy metabolism, 77 genes involved in lipid metabolism, and 85 genes linked to nucleotide metabolism. Comparative analysis with closely related species (*R. corynebacterioides* DSM 20151ᵀ, *R. trifolii* T8ᵀ, and *R. kroppenstedtii* DSM 44908ᵀ ) revealed both shared and distinct metabolic features. While the overall distribution of major metabolic categories was broadly comparable across the four genomes, strain FMA22ᵀ exhibited moderate differences in several pathways. For instance, genes related to xenobiotics biodegradation and metabolism were less abundant in FMA22^T^ (62 genes) compared to *R*. *trifolii* T8ᵀ (127 genes), suggesting possible ecological divergence in substrate utilization capacity. Similarly, minor quantitative variations were observed in pathways associated with carbohydrate metabolism, amino acid metabolism, and secondary metabolite biosynthesis. However, the overall functional profile of strain FMA22^T^ remains consistent with the metabolic versatility characteristic of the genus *Rhodococcus*. Taken together, KEGG-based functional annotation indicates that strain FMA22^T^ possesses a comprehensive metabolic framework, while also displaying subtle pathway-level distinctions that may reflect ecological adaptation to the mining soil environment.

RAST and SEED Viewer annotation revealed that strain FMA22^T^ possessed a broad functional repertoire distributed across major subsystem categories. The overall subsystem coverage was approximately 22%, indicating a substantial proportion of genes assigned to curated functional subsystems. Among the annotated categories, the most represented functional groups in strain FMA22^T^ were amino acids and derivatives (295 CDSs), carbohydrates (204 CDSs), cofactors, vitamins, prosthetic groups and pigments (157 CDSs), protein metabolism (76 CDSs), fatty acids, lipids, and isoprenoids (127 CDSs), and stress response (32 CDSs) (Fig. S14; Table S4). These categories collectively reflect the core metabolic and adaptive capacities observed in strain FMA22^T^ and its closest phylogenetic relatives. Comparative analysis with *R. corynebacterioides* DSM 20151ᵀ, *R. trifolii* T8ᵀ, and *R. kroppenstedtii* DSM 44908ᵀ demonstrated broadly similar subsystem distributions, consistent with their phylogenetic relatedness. However, quantitative differences were observed in several functional categories. Strain FMA22^T^ exhibited a relatively higher number of genes associated with amino acid metabolism and carbohydrate metabolism compared to R. *corynebacterioides* DSM 20151ᵀ, whereas membrane transport and regulatory functions showed minor variations among the compared genomes. Notably, genes assigned to the category “cofactors, vitamins, prosthetic groups and pigments” were well represented in strain FMA22^T^ (157 CDSs), supporting its metabolic versatility and potentially contributing to pigment biosynthesis capacity. Additionally, the presence of stress response genes and membrane transport systems indicates the ability of strain FMA22^T^ to adapt to fluctuating environmental conditions, consistent with its isolation from a metal-rich mining environment.

### Phenotypic and chemotaxonomic characterization

Strain FMA22^T^ formed circular, convex and orange-red colonies on NA and exhibited aerobic, non-sporeforming and non-motile cell morphology. The strain showed growth at 4–40 °C (optimum at 25 °C), pH 5.0–9.0 (optimum pH 7.0) and in the presence of up to 3% NaCl (optimal at 0%, w/v), indicating that NaCl is not necessary for its growth. Strain FMA22^T^ and the related type strains *R. corynebacterioides* DSM 20151ᵀ, *R. kroppenstedtii* DSM 44908ᵀ, and *R. trifolii* T8ᵀ were negative for oxidase activity. In the VITEK 2 GP card, the strain FMA22^T^ and the reference strains were positive for L-proline arylamidase, and arginine dihydrolase but negative for phosphatidylinositol phospholipase C, α-galactosidase, α-glucosidase, β-galactosidase, β-glucuronidase and β-galactopyranosidase. According to the API 50 CH test, the strain FMA22^T^ and the reference strains produced acid from D-sucrose and D-fructose, but not from inulin, D-maltose, D-mannitol, D-turanose, D-lyxose, L-xylose. In API 20E, the type strain and the reference strains utilized citrate as the sole carbon source and did not produce hydrogen sulfide, indole or acetoin.

Although strain FMA22^T^ shared several core phenotypic traits with its closest phylogenetic relatives (*R. corynebacterioides* DSM 20151ᵀ, *R. trifolii* T8ᵀ, and *R. kroppenstedtii* DSM 44908ᵀ), it exhibited distinct differential characteristics (Table 3). Strain FMA22^T^ differed from the reference strains in its carbohydrate utilization profile. It was positive for acid production from L-arabinose and gentibiose, whereas these reactions varied among the related species. In contrast, FMA22^T^ did not produce acid from D-galactose, D-cellobiose, D-mannitol, D-mannose, D-ribose, D-sorbitol, or D-xylose, while one or more of the reference strains were positive for these substrates. Growth characteristics also distinguished strain FMA22ᵀ. It grew at temperatures ranging from 4 to 40 °C, with an optimum at 25 °C, which is lower than the optimal temperatures observed for the related strains (30–35 °C). Furthermore, FMA22^T^ tolerated NaCl concentrations up to 3% (w/v), whereas *R. corynebacterioides* DSM 20151ᵀ, *R. kroppenstedtii* DSM 44908ᵀ, and *R. trifolii* T8ᵀ tolerated higher salt concentrations (up to 6–9% NaCl). Enzymatic activity profiles further differentiated strain FMA22^T^. The strain was positive for α-glucosidase, arginine dihydrolase, alanine arylamidase, tyrosine arylamidase, leucine arylamidase, Ala-Phe-Pro arylamidase, and urease, whereas the presence or absence of these activities varied among the reference strains. Notably, FMA22^T^ lacked phosphatase and α-mannosidase activities, which were detected in some of the related species.

**Table 3:** Differential phenotypic characteristics of strain FMA22^T^ and its closest phylogenetic relatives. (1) FMA22^T^; (2) *R. corynebacterioides* DSM 20151ᵀ; (3) *R. kroppenstedtii* DSM 44908ᵀ; (4) *R. trifolii* T8ᵀ. All data were obtained in this study.

Characteristic	1	2	3	4
Acid production from				
D-Cellobiose	-	+	+	+
D-Galactose	-	+	-	+
D-lyxose	-	+	+	-
D-Mannitol	-	+	+	+
D-Mannose	-	+	+	-
D-raffinose	-	-	+	-
D-Ribose	-	+	+	-
D-Sorbitol	-	+	+	+
D-trehalose	-	+	+	+
D-turanose	-	-	+	+
D-Xylose	-	+	+	-
Gentibiose	+	+	-	-
L-Arabinose	+	-	+	+
L-rhamnose	+	-	-	-
Methyl-alpha-D-glucopyranoside	-	-	-	+
Potassium 2- ketogluconate	-	+	+	-
Salicin	-	-	-	+
Xylitol	+	+	-	-
Fermentation (API 20E):				
Inositol	-	+	+	+
Melibiose	-	+	-	+
Amygdalin	-	+	+	-
Growth for				
Temperature range (°C)	4–40	22–45	10–37	15–50
Optimal temperature (°C)	25	30	30	35
Optimal pH	7.0	7.5	8.0	7.5
NaCl range (w/v, %)	0–3	0–7	0–9	0–6
Enzyme activity				
Alpha glucosidase	+	-		
Arginine dihydrolase	+	-	+	-
Phosphatase	-	-	+	-
Alanine arylamidase	+	+	-	+
Tyrosine arylamidase	+	-	+	-
Leucine arylamidase	+	+	+	-
Ala-Phe-Pro arylamidase	+	+	-	+
L-aspartate arylamidase	-	-	-	+
Alpha-mannosidase	-	+	+	-
L-pyrrolidonyl-arylamidase	-	-	-	+
Urease	+	-	-	+
Nitrate reduction	-	+	-	-

The cellular fatty acid profile of strain FMA22^T^ was consistent with members of the genus *Rhodococcus*, while displaying quantitative differences compared with its closest phylogenetic relatives (Table S5). The major fatty acids (> 10%) of strain FMA22^T^ were C18:1 ω9c (26.64%), summed feature 3 (21.83%; comprising C16:1 ω7c / C16:1 ω6c), and C16:0 (18.87%). In addition, 10-methyl C18:0 was present at a relatively high level (9.56%), representing a notable component of the profile. In comparison, *R. corynebacterioides* DSM 20151ᵀ exhibited C16:0 (21.5%) and C18:1 ω9c (22.8%) as its predominant fatty acids, but contained a markedly lower proportion of summed feature 3 (13.1%) than strain FMA22^T^. *R. kroppenstedtii* DSM 44908ᵀ was characterized by a substantially higher proportion of C16:0 (28.5%) and a reduced level of summed feature 3 (10.7%). Similarly, *R. trifolii* T8ᵀ displayed lower C18:1 ω9c (19.1%) and summed feature 3 (15.7%) compared with FMA22ᵀ, but showed a higher proportion of 10-methyl C18:0 (11.9%). Minor but consistent differences were also observed in anteiso-C15:0, C17:1 ω8c, and 10-methyl C17:0 among the compared strains. Overall, while the fatty acid composition of strain FMA22^T^ conforms to the chemotaxonomic characteristics of the genus *Rhodococcus*, the observed quantitative differences in major components further support its phenotypic distinction from closely related species.

**Fig. 3:**
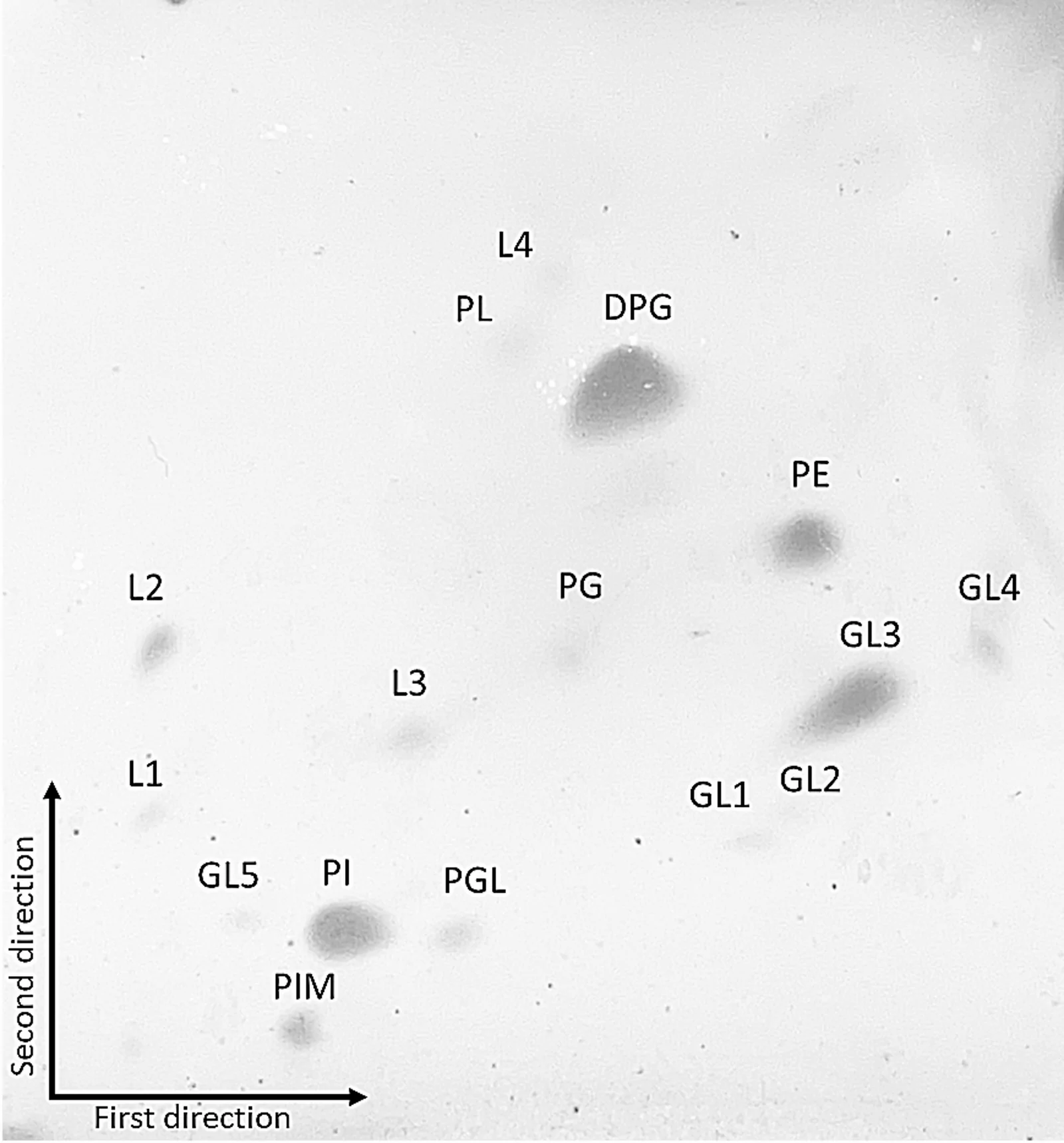
Total polar lipid profiles of strain FMA22^T^. Abbreviations: PE, phosphatidylethanolamine; PG, phosphatidylglycerol; DPG, diphosphatidylglycerol; PC, phosphatidylcholine; PI, phosphatidylinositol; PIM, phosphatidylinositol mannoside; GL, unidentified glycolipid; PL, unidentified phospholipid; L, unidentified lipids; PGL, unidentified phosphoglycolipid.

The polar lipids of strain FMA22^T^ consisted of phosphatidylethanolamine, diphosphatidylglycerol, phosphatidylinositol, phosphatidylinositol mannoside, phosphatidylcholine, five unidentified glycolipids, four unidentified lipids, an unidentified phospholipid and an unidentified phosphoglycolipid (Fig. 3). MK-8(H_2_) was identified as the major isoprenoid quinone of strain FMA22^T^, consistent with those reported for closely related species *R. corynebacterioides* DSM 20151ᵀ, *R. trifolii* T8ᵀ, and *R. kroppenstedtii* DSM 44908ᵀ^58–60^. Cell wall analysis demonstrated the presence of meso-diaminopimelic acid (meso-A2pm) as the diagnostic diamino acid, together with arabinose and galactose as characteristic whole-cell sugars. This composition corresponds to cell wall chemotype IV according to Lechevalier & Lechevalier (1970)^61^ and is typical for members of the genus *Rhodococcus*.

### Production and characterization of the orange-red pigment

Strain FMA22^T^ produced orange-red-pigmented colonies under the tested culture conditions. The methanolic extract exhibited a characteristic absorption maximum at 475 nm in the UV-VIS spectrum, consistent with conjugated polyene chromophores typically observed in carotenoid-type pigments^62^.

Genome-based analyses were consistent with the possible carotenoid nature of the pigment. KEGG pathway annotation identified genes involved in terpenoid and polyketide metabolism (30 genes) and confirmed the presence of the carotenoid biosynthesis pathway. In particular, the key carotenoid biosynthetic genes *crtB* (phytoene synthase), *crtI* (phytoene desaturase), and *crtY* (lycopene cyclase) were detected, indicating a functional pathway for the conversion of geranylgeranyl pyrophosphate into cyclic carotenoid end-products. Consistent with these findings, antiSMASH analysis revealed a terpene biosynthetic gene cluster annotated as a carotenoid-type cluster in strain FMA22^T^. RAST subsystem analysis further identified genes associated with cofactors, vitamins, prosthetic groups, and pigments, corroborating the metabolic framework for carotenoid biosynthesis. Carotenoid production is a well-recognized trait within the genus *Rhodococcus*, where these pigments are frequently associated with oxidative stress tolerance and protection against photooxidative damage. The presence of a complete crt gene set and a terpene biosynthetic cluster in FMA22^T^ therefore not only explains its distinct orange-red pigmentation but may also contribute to cellular stability under environmental stress conditions. Taken together, the combined spectroscopic and genomic evidence suggests that strain FMA22^T^ produces a carotenoid-type orange-red pigment and possesses a putative carotenoid biosynthetic pathway.

### Antioxidant activities

**Fig. 4:**
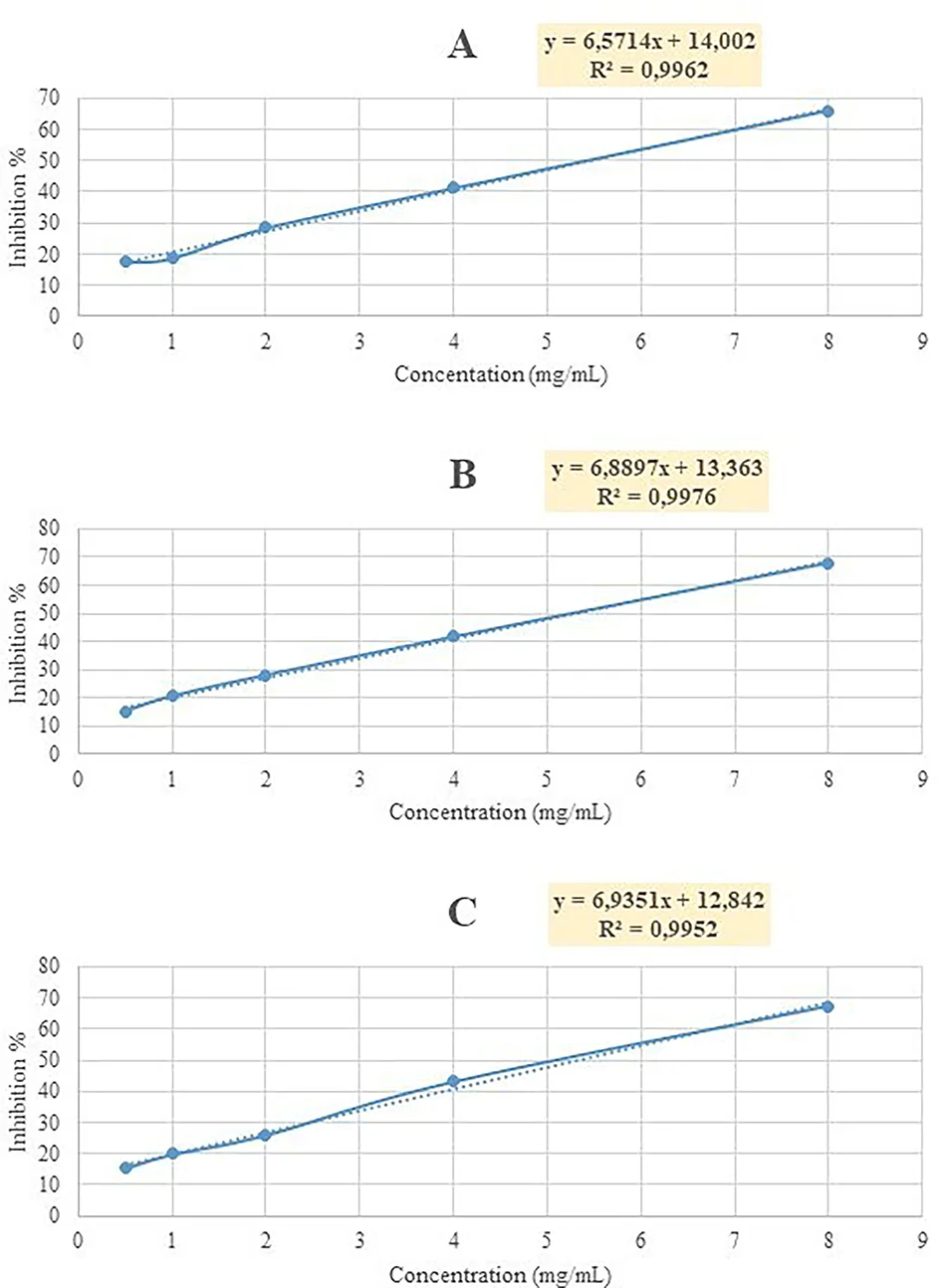
DPPH radical scavenging activity of the orange-red pigment extract obtained from strain FMA22^T^ at different concentrations. Panels (A-C) represent three independent experimental replicates used for SC₅₀ determination. Linear regression equations and correlation coefficients (R²) are shown for each replicate. Data variability among triplicate measurements was low, supporting the reproducibility of the antioxidant activity results.

The orange-red pigment extract obtained from strain FMA22^T^ exhibited a concentration-dependent DPPH radical scavenging activity. A gradual increase in inhibition percentage was observed with rising extract concentrations, indicating the presence of compounds capable of neutralizing free radicals (Fig. 4). All measurements were performed in triplicate, and the results were expressed as mean ± standard deviation (SD). The dose-response analysis yielded an SC₅₀ value of 5.38 ± 0.08 mg/mL, reflecting a measurable antioxidant capacity of the extract. Trolox was employed as the standard reference antioxidant and demonstrated an SC₅₀ value of 6.81 ± 0.29 µg/mL. Considering the substantial difference in magnitude between the two values, Trolox showed markedly higher radical-scavenging efficiency, as expected from a pure synthetic antioxidant. Since Trolox was included as a reference standard rather than a comparative experimental group, no further statistical comparison was performed between Trolox and the extract. Although the antioxidant activity of the extract was considerably lower than that of Trolox, the observed concentration-dependent radical scavenging activity still supports the presence of bioactive secondary metabolites capable of participating in hydrogen atom transfer or single-electron transfer mechanisms. The antioxidant performance of the FMA22^T^ pigment extract may therefore be considered measurable, particularly considering that the extract represents a crude natural microbial metabolite mixture rather than a purified antioxidant compound.

Evaluation through the FRAP method further confirmed the reducing ability of the orange-red pigment extract. Using the Trolox calibration curve as a standard reference, the ferric reducing activity of the extract was calculated as 4.34 ± 0.308 µmol TE/g extract. This outcome demonstrates that the extract can effectively participate in electron-transfer processes, facilitating the conversion of Fe³⁺ to Fe²⁺ within the assay system. The relatively low variability between replicate measurements supports the reproducibility of the reducing capacity. The antioxidant activity observed in the present study is consistent with previous reports describing microbial pigments with radical scavenging and reducing properties. Comparable studies on pigment-producing microorganisms have reported that crude microbial pigment extracts may exhibit detectable DPPH radical scavenging and ferric reducing activities depending on pigment composition and metabolite profile. In a recent study, similarly reported measurable antioxidant activity in microbial pigment extracts, although their activities remained lower than those of pure synthetic antioxidant standards^63^. In agreement with these findings, the results obtained for strain FMA22^T^ suggest the presence of redox-active metabolites with potential biological functionality.

### HIV-1 reverse transcriptase inhibition activity

The inhibitory activity of the orange-red pigment extracted from strain FMA22^T^ against HIV-1 Reverse Transcriptase (HIV-1 RT) was evaluated using a non-radioactive colorimetric assay. The pigment extract exhibited concentration-dependent inhibition of RT activity, with an IC₅₀ value of 22 mg mL⁻¹ (Fig. S15). Although this IC₅₀ value does not indicate strong inhibition compared to clinically used RT inhibitors, it demonstrates weak but measurable anti-RT activity. Since the tested material represents a crude methanolic pigment extract rather than a purified compound, molar concentration could not be calculated.

Microbial pigments have been investigated for antiviral properties in various systems. Among bacterial pigments, the indole-derived compound violacein has been reported to exhibit in vitro inhibitory activity against HIV-1 RT^13^. However, such studies are largely limited to non-carotenoid pigments. With respect to carotenoids, direct HIV-1 RT inhibition studies are scarce. One of the few reported examples is halocynthiaxanthin, a carotenoid associated with the marine tunicate *Halocynthia roretzi*, which has been shown to inhibit HIV-1 and HIV-2 RT at micromolar concentrations^64^. Nevertheless, this compound is not of bacterial origin, and evidence for HIV-1 RT inhibition by bacterial carotenoid pigments remains extremely limited.

To date, no studies have evaluated HIV-1 RT inhibition specifically in carotenoid pigments derived from the genus *Rhodococcus*. In this context, the measurable inhibitory activity observed for the orange-red pigment of strain FMA22ᵀ represents a novel preliminary finding. While the IC₅₀ value indicates weak inhibitory activity, the results suggest that bacterial carotenoid-type pigments from mining-associated environments may exhibit detectable interaction with HIV-1 RT at the enzyme level, warranting further purification and structural characterization studies.

## Conclusion

This study reports the comprehensive taxonomic and functional characterization of strain FMA22^T^, isolated from mining-impacted soil in Türkiye. A polyphasic taxonomic approach integrating phenotypic, chemotaxonomic, and genome-based analyses demonstrated that strain FMA22^T^ represents a novel species within the genus *Rhodococcus*. Phylogenomic reconstruction (UBCG2 and TYGS) together with ANI, AAI, and dDDH values confirmed its clear genomic separation from its closest relatives. The type strain FMA22^T^ has been deposited in the Belgian Coordinated Collections of Microorganisms (BCCM/LMG) and the Deutsche Sammlung von Mikroorganismen und Zellkulturen (DSMZ) under the accession numbers LMG 34,144^T^ and DSM 120,048^T^, respectively.

Genome analysis revealed a diverse biosynthetic repertoire, including carotenoid-associated terpene clusters, NRPS- and PKS-related pathways, and stress-adaptation genes consistent with survival in metal-rich and oxidative environments. The strain produces a distinct orange-red carotenoid-type pigment supported by spectroscopic and genomic evidence. Functional assays demonstrated measurable antioxidant capacity and concentration-dependent inhibition of HIV-1 reverse transcriptase activity.

Together, these findings expand the known diversity of the genus *Rhodococcus* and identify strain FMA22^T^ as a metabolically versatile actinobacterium with measurable antioxidant activity and detectable enzyme-level interaction with HIV-1 RT. Further purification and structural elucidation of its pigment-associated metabolites will clarify their biological relevance and mechanism of action.

### Description of *Rhodococcus folensis* sp. nov.

*Rhodococcus folensis* (fo.len′sis N.L. masc. adj. *folensis*, pertaining to Fol, the former name historically used for both Vakfıkebir district and Kalınçam village in Trabzon, Türkiye, where the type strain was isolated).

Cells are Gram-stain-positive, aerobic, non-sporeforming and non-motile. Colonies grown on NA at 25 °C for two days are circular, convex, and orange-red. Growth occurs at 4–40 °C (optimum 25 °C), pH 5.0–9.0 (optimum 7.0), and in the presence of 0–3% (w/v) NaCl. Nitrate is not reduced to nitrite. Produce Ala-Phe-Pro arylamidase, alanine arylamidase, arginine dihydrolase, catalase, L-proline arylamidase, leucine arylamidase, ornithine decarboxylase, tyrosine arylamidase and urease, but not α-galactosidase, α-mannosidase, β-galactopyranosidase, β-galactosidase, β-glucuronidase, gelatinase, L-aspartate arylamidase, L-pyrrolidonyl arylamidase, lysine decarboxylase, oxidase, phosphatase, phosphatidylinositol phospholipase C, tryptophan deaminase and tryptophanase. Produce acid from D-fructose, D-fucose, D-glucose, D-sucrose, gentibiose, L-arabinose, L-rhamnose and xylitol, but not from amygdalin, arbutin, D-arabinose, D-cellobiose, D-galactose, D-lactose, D-lyxose, D-maltose, D-mannitol, D-mannose, D-melezitose, D-melibiose, D-raffinose, D-ribose, D-sorbitol, D-tagatose, D-trehalose, D-turanose, D-xylose, D/L-arabitol, D/L-fucose, erythritol, esculin ferric citrate, glycerol, glycogen, inositol, inulin, L-sorbose, L-xylose, methyl-α-D-glucopyranoside, methyl-α-D-mannopyranoside, methyl-β-D-xylopyranoside, N-acetyl-glucosamine, potassium 2/5-ketogluconate, pullulan and starch. Utilize citrate as sole carbon source. Do not alkalinize L-lactate. Hydrogen sulfide, indole and acetoin are not produced. Major fatty acids were C18:1 ω9c, summed feature 3 (comprising C16:1 ω7c / C16:1 ω6c), and C16:0. The predominant respiratory quinone is MK-8(H2) and the polar lipid consists of phosphatidylethanolamine, diphosphatidylglycerol, phosphatidylinositol, phosphatidylinositol mannoside, phosphatidylcholine, five unidentified glycolipids, four unidentified lipids, an unidentified phospholipid and an unidentified phosphoglycolipid. The genome size is 4.2 Mb with a genomic DNA G + C content of 67%.

The type strain FMA22^T^ (= LMG 34144^T^ =DSM 120048^T^) was isolated from a soil sample collected at an abandoned mining site in Kalınçam village, Vakfıkebir district, Trabzon, Türkiye. The 16 S rRNA gene for the strain FMA22^T^ has been assigned the GenBank accession number PZ092762. The Whole Genome Shotgun project has been deposited at DDBJ/ENA/GenBank under the accession PRJNA1425433.

## Supplementary Information

Below is the link to the electronic supplementary material.

Supplementary Material 1

## Data Availability

The 16 S rRNA gene sequences of strain FMA22ᵀ obtained by Sanger sequencing (1441 bp) and extracted from the genome assembly (1525 bp) have been deposited in GenBank/EMBL/DDBJ under accession numbers PZ033343 and PZ092762, respectively. The Whole Genome Shotgun project is available under BioProject accession number PRJNA1425433.
