# Supplementary material for: Rhodococcus folensis sp. nov., an orange-red-pigmented bacterium from mining soil
Source: Sci Rep. 2026 Jun 21;16:28374. doi: 10.1038/s41598-026-58503-0 (PMC13562592; doi:10.1038/s41598-026-58503-0)
Supplement: Supplementary file 1 [file 41598_2026_58503_MOESM1_ESM.docx]

**Supplementary data**

***Rhodococcus folensis* sp. nov., an orange-red-pigmented bacterium from mining soil**

Aleyna NALCAOGLU SENOCAK^1,2^, Omer Faruk OZTURK^1,2^, Miray KILIC^1,2^, Ersan BEKTAS^3^, Ali Osman BELDUZ^4^, Kadriye INAN BEKTAS^1^*

^1^Karadeniz Technical University, Faculty of Science, Department of Molecular Biology and Genetics, 61080 Trabzon, Türkiye

^2^Karadeniz Technical University, Graduate School of Natural and Applied Science, Department of Molecular Biology and Genetics, 61080 Trabzon, Turkey

^3^Espiye Vocational School, Giresun University, Giresun, Turkey

^3^Karadeniz Technical University, Faculty of Science, Department of Biology, 61080 Trabzon, Türkiye

* e-mail: kadriyensis@gmail.com, phone: +904623773687

***Corresponding author:**

Kadriye İNAN BEKTAS

**Article type**: Taxonomic description

**Contents of Supplementary Materials**

1. Supplementary figures S1-S15 with figure legends

2. Supplementary tables S1-S5 with table legends

**Fig. S1** Comparative colony morphology of Rhodococcus folensis FMA22ᵀ and closely related reference strains grown on agar medium. A) FMA22^T^; B) *R.* *corynebacterioides* DSM 20151ᵀ; C) *R.* *kroppenstedtii* DSM 44908ᵀ; D) *R.* *trifolii* T8^T^

**Fig. S2** The relationship between strain FMA1 and other related type strains are depicted in this maximum-parsinomy phylogenetic tree, which is based on 16S rRNA gene sequences. *Macrococcoides canis* was used as an outgroup. Bar, 0.01 substitutions per nucleotide position

**Fig. S3** The relationship between strain FMA9 and other related type strains are depicted in this maximum-parsinomy phylogenetic tree, which is based on 16S rRNA gene sequences. *Nocardia vaccinii* was used as an outgroup. Bar, 0.01 substitutions per nucleotide position

**Fig. S4** The relationship between strain FMAX and other related type strains are depicted in this maximum-parsinomy phylogenetic tree, which is based on 16S rRNA gene sequences. *Kytococcus sedentarius* was used as an outgroup. Bar, 0.01 substitutions per nucleotide position

**Fig. S5** The relationship between strain UGA5 and other related type strains are depicted in this maximum-parsinomy phylogenetic tree, which is based on 16S rRNA gene sequences. *Cellvibrio japoninus* was used as an outgroup. Bar, 0.01 substitutions per nucleotide position

**Fig. S6** The relationship between strain UGA11 and other related type strains are depicted in this maximum-parsinomy phylogenetic tree, which is based on 16S rRNA gene sequences. Bar, 0.01 substitutions per nucleotide position

**Fig. S7** The relationship between strain UGA12 and other related type strains are depicted in this maximum-parsinomy phylogenetic tree, which is based on 16S rRNA gene sequences. *Alkalihalobacillus alcalophilus* was used as an outgroup. Bar, 0.01 substitutions per nucleotide position

**Fig. S8** The relationship between strain FMA23 and other related type strains are depicted in this maximum-parsinomy phylogenetic tree, which is based on 16S rRNA gene sequences. *Skermania pinensis* was used as an outgroup. Bar, 0.01 substitutions per nucleotide position

**Fig. S9** The relationship between strain FMA22^T^ and other related type strains are depicted in this maximum-parsinomy phylogenetic tree, which is based on 16S rRNA gene sequences. Bootstrap values exceeding 50% are indicated at the branch points. *Micromonospora aurantiaca* ATCC 27029^T^ was used as an outgroup.

**Fig. S10** The relationship between strain FMA22^T^ and other related type strains are depicted in this neighbour-joining phylogenetic tree, which is based on 16S rRNA gene sequences. Bootstrap values exceeding 50% are indicated at the branch points. *Micromonospora aurantiaca* ATCC 27029^T^ was used as an outgroup. Bar, 0.01 substitutions per nucleotide position

**Fig. S11** Phylogenetic tree based on whole-genome sequences of strain FMA22^T^ and related reference strains. The tree was inferred with FastME 2.1.6.1 from genome blast distance phylogeny (GBDP) distances calculated from genome sequences using the TYGS server (https:// tygs.dsmz.de). The branch lengths are scaled in terms of GBDP distance formula d5. The numbers at branches are GBDP pseudo-bootstrap support values ≥64% from 100 replications with an average branch support of 97.7%. The tree was rooted at the midpoint.

**Fig. S12** Comparative antiSMASH analysis of carotenoid-related biosynthetic gene clusters identified in Rhodococcus folensis FMA22ᵀ and closely related Rhodococcus species. A) FMA22^T^; B) *R.* *corynebacterioides* DSM 20151ᵀ; C) *R.* *kroppenstedtii* DSM 44908ᵀ; D) *R.* *trifolii* T8^T^

**Fig. S13** Comparative heatmap analysis of carotenoid-related proteins among Rhodococcus folensis FMA22ᵀ and closely related Rhodococcus species.

**Fig. S14** Visualization of genomic data and analysis of biosynthetic gene clusters: Seed Viewer results. Comparative genomic analyses of strains; A) FMA22^T^; B) *R.* *corynebacterioides* DSM 20151ᵀ; C) *R.* *kroppenstedtii* DSM 44908ᵀ; D) *R.* *trifolii* T8^T^ were performed using the SEED Viewer.

**Fig. S15** HIV-1 Reverse Transcriptase (RT) inhibitory activity of the crude carotenoid extract obtained from Rhodococcus folensis FMA22ᵀ.

**Table S1.** Pigmented bacterial isolates obtained from environmental samples and their closest phylogenetic relatives based on 16S rRNA gene sequence analysis.

| **Strain** | **Colony Color** | **Closest Type Strain (16S rRNA)** | **Similarity (%)** |
| --- | --- | --- | --- |
| FMA22^T^ | Orange–red | *Rhodococcus corynebacterioides* | %99.57 |
| FMAX | Dull yellow | *Micrococcus luteus* | %99.63 |
| FMA9 | Yellowish-brown | *Pseudonocardia carboxydivorans* | %100 |
| UGA11 | Yellow | *Plantibacter flavus* | %99.93 |
| FMA23 | Dark orange | *Williamsia muralis* | %99.63 |
| UGA12 | Orange | *Exiguobacterium sibiricum* | %99.79 |
| FMA 1 | Dark yellow | *Staphylococcus hominis* subsp. *hominis* | %99.86 |
| UGA5 | Bright yellow | *Pseudomonas sivasensis* | %100 |

**Table S2.** Carbohydrate active enzymes (CAZymes) annotation results of strain FMA22^T^ and its closest phylogenetic relative. Carbohydrate active enzymes (CAZymes); glycosyltransferases (GTs), glycoside hydrolases (GHs), carbohydrate-binding modules (CBMs), polysaccharide lyases (PLs), auxiliary activities (AAs) and carbohydrate esterases (CEs)

|  | **GT** | **GH** | **CBM** | **CE** | **AA** |
| --- | --- | --- | --- | --- | --- |
| *R.* *kroppenstedtii* DSM 44908ᵀ | 57 | 35 | 6 | 21 | 5 |
| *R.* *trifolii* T8ᵀ | 69 | 42 | 5 | 21 | 11 |
| *R.* *corynebacterioides* DSM 20151ᵀ | 56 | 31 | 5 | 10 | 6 |
| FMA22^T^ | 60 | 37 | 5 | 12 | 7 |

**Table S3.** Numbers of genes associated with the KEGG functional pathways.

1.FMA22^T^; 2. *R.* *corynebacterioides* DSM 20151ᵀ; 3. *R.* *kroppenstedtii* DSM 44908ᵀ*;* 4. *R.* *trifolii* T8ᵀ.

| **Obtain from KEGG pathways** | **1** | **2** | **3** | **4** |
| --- | --- | --- | --- | --- |
| **Metabolism** |  |  |  |  |
| Global and overview maps | 1539 | 1452 | 1449 | 1692 |
| Carbohydrate metabolism | 245 | 231 | 226 | 275 |
| Energy metabolism | 131 | 129 | 129 | 140 |
| Lipid metabolism | 77 | 76 | 73 | 81 |
| Nucleotide metabolism | 85 | 66 | 70 | 87 |
| Amino acid metabolism | 240 | 211 | 212 | 266 |
| Metabolism of other amino acids | 52 | 39 | 41 | 63 |
| Glycan biosynthesis and metabolism | 89 | 88 | 86 | 91 |
| Metabolism of cofactors and vitamins | 166 | 149 | 158 | 171 |
| Metabolism of terpenoids and polyketides | 30 | 29 | 29 | 30 |
| Biosynthesis of other secondary metabolites | 38 | 36 | 37 | 43 |
| Xenobiotics biodegradation and metabolism | 62 | 76 | 53 | 127 |
| **Genetic Information Processing** |  |  |  |  |
| Transcription | 5 | 4 | 5 | 5 |
| Translation | 78 | 78 | 78 | 78 |
| Folding, sorting and degradation | 41 | 40 | 41 | 42 |
| Replication and repair | 69 | 76 | 68 | 69 |
| Information processing in viruses | 1 | 1 | 1 | 1 |
| **Environmental Information Processing** |  |  |  |  |
| Membrane transport | 103 | 94 | 102 | 106 |
| Signal transduction | 57 | 60 | 58 | 62 |
| **Cellular Processes** |  |  |  |  |
| Transport and catabolism | 12 | 11 | 12 | 15 |
| Cell growth and death | 16 | 16 | 16 | 16 |
| Cell motility | 3 | 2 | 2 | 5 |
| Cellular community - prokaryotes | 41 | 44 | 43 | 47 |
| **Organismal Systems** |  |  |  |  |
| Aging | 12 | 12 | 12 | 12 |
| Environmental adaptation | 6 | 6 | 6 | 6 |
| Immune system | 5 | 5 | 5 | 5 |
| Endocrine system | 19 | 18 | 18 | 18 |
| Digestive system | 11 | 11 | 11 | 11 |
| Excretory system | 2 | 2 | 2 | 2 |
| Nervous system | 6 | 7 | 6 | 7 |
| Development and regeneration | 1 | 1 | 1 | 1 |
| **Human Diseases** |  |  |  |  |
| Cancer: overview | 17 | 16 | 17 | 17 |
| Cancer: specific types | 4 | 4 | 4 | 4 |
| Infectious disease: bacterial | 14 | 13 | 13 | 14 |
| Infectious disease: parasitic | 2 | 2 | 2 | 2 |
| Infectious disease: viral | 1 | 1 | 1 | 1 |
| Immune disease | 2 | 2 | 2 | 2 |
| Neurodegenerative disease | 14 | 14 | 16 | 14 |
| Cardiovascular disease | 10 | 10 | 10 | 10 |
| Substance dependence | 3 | 3 | 3 | 3 |
| Endocrine and metabolic disease | 10 | 10 | 9 | 11 |
| Drug resistance: antimicrobial | 15 | 16 | 16 | 16 |
| Drug resistance: antineoplastic | 6 | 6 | 6 | 6 |

**Table S4.** Comparative RAST subsystem distribution analysis of Rhodococcus folensis FMA22ᵀ and closely related Rhodococcus species.

1.FMA22^T^; 2. *R.* *corynebacterioides* DSM 20151ᵀ; 3. *R.* *kroppenstedtii* DSM 44908ᵀ*;* 4. *R.* *trifolii* T8ᵀ.

|  | **1** | **2** | **3** | **4** |
| --- | --- | --- | --- | --- |
| Fatty Acids, Lipids and Isoprenoids | 100 | 99 | 127 | 99 |
| Phosphorus Metabolism | 20 | 20 | 23 | 20 |
| Phages, Prophages, Transposable elements, Plasmids | 4 | 0 | 3 | 4 |
| Nodulation | 0 | 0 | 0 | 0 |
| Regulation and Cell signaling | 14 | 13 | 14 | 14 |
| Metabolism of Aromatic Compounds | 10 | 26 | 60 | 19 |
| Membrane Transport | 28 | 18 | 35 | 23 |
| Cofactors, Vitamins, Prostheti Groups, Pigments | 147 | 134 | 172 | 141 |
| Stress Response | 27 | 27 | 32 | 26 |
| Virulence, Disease and Defence | 24 | 23 | 31 | 24 |
| Respiration | 75 | 76 | 90 | 74 |
| Secondary Metabolism | 4 | 3 | 8 | 4 |
| Cell Wall and Capsule | 32 | 23 | 32 | 24 |
| DNA Metabolism | 81 | 77 | 84 | 94 |
| Amino Acisd and Derivates | 295 | 237 | 333 | 249 |
| Dormancy and Sporulation | 1 | 1 | 1 | 1 |
| Photosynthesis | 0 | 0 | 0 | 0 |
| Nitrogen Metabolism | 16 | 9 | 20 | 9 |
| Nucleosides and Nucteotides | 86 | 49 | 103 | 64 |
| Miscellaneous | 30 | 31 | 38 | 27 |
| RNA Metabolism | 34 | 33 | 39 | 33 |
| Cell Division and Cell Cycle | 0 | 0 | 0 | 0 |
| Carbonhydrates | 204 | 154 | 279 | 169 |
| Iron acquisition and Metabolism | 3 | 2 | 3 | 3 |
| Potassium Metabolism | 7 | 7 | 7 | 7 |
| Protein Metabolism | 169 | 167 | 171 | 170 |
| Sulfur Metabolism | 5 | 5 | 7 | 7 |
| Motility and Chemotaxis | 0 | 0 | 0 | 0 |

**Table S5.** Cellular fatty acid profiles of strain FMA22^T^ and its closest phylogenetic relative.

1.FMA22^T^; 2. *R.* *corynebacterioides* DSM 20151ᵀ; 3. *R.* *kroppenstedtii* DSM 44908ᵀ*;* 4. *R.* *trifolii* T8ᵀ.

All data were obtained in this study. Fatty acids amounting to more than 10% are highlighted in bold. –, not detected.

| **Fatty acid** | **1** | **2** | **3** | **4** |
| --- | --- | --- | --- | --- |
| C_14:0_ | 2.79 | 4.8 | 5.2 | 6.5 |
| C_15:1_ ω5*c* | 1.0 | 0.9 | 0.8 | 0.6 |
| C_15:0_ | - | - | - | 3.0 |
| anteiso-C_15:0_ | 3.63 | 1.2 | 0.8 | 0.6 |
| C_16:0_ | **18.87** | **21.5** | **28.5** | **21.6** |
| C_16:0_ 2OH | 0.58 | - | - | - |
| anteiso-C_16:0_ | 1.32 | 0.6 | 0.8 | 0.5 |
| C_17:1_ ω8*c* | 7.60 | 8.1 | 9.6 | 5.4 |
| C_17:0_ | 1.12 | 0.9 | 2.0 | 1.1 |
| 10-methyl C_17:0_ | 1.45 | 0.5 | 1.7 | 2.1 |
| C_18:1_ ω9*c* | **26.64** | **22.8** | **26.8** | **19.1** |
| 10-methyl C_18:0_ | 9.56 | 8.7 | 5.6 | **11.9** |
| Summed feature 3 | **21.83** | **13.1** | **10.7** | **15.7** |
| Summed feature 6 | 1.08 | 1.0 | 1.5 | 3.2 |

* Summed features consist of one or more fatty acids that cannot be separated by the method used. Summed feature 3 contains C16:1 ω7c / C16:1 ω6c; summed feature 6 contains C19:1 ω11c / C19:1 ω9c.
